# The complete mitochondrial genome of West Indian drywood termite, *Cryptotermes brevis* (Walker) (Isoptera: Kalotermitidae)

**DOI:** 10.1080/23802359.2019.1624212

**Published:** 2019-07-11

**Authors:** Peishan He, Quan Zhou, Kaiping Hu, Ting Li, Yang Wen, Ling Zhang, Jianguo Wang

**Affiliations:** School of Agricultural Sciences, Jiangxi Agricultural University, Nanchang, P. R. China

**Keywords:** **M**itochondrial genome, *Cryptotermes brevis*, phylogenetic tree

## Abstract

The complete mitogenome of *Cryptotermes brevis* (Walker) (Isoptera: Kalotermitidae) is confirmed. This circular mitogenome (accession number MK618724) length is 15,674 bp, containing 13 protein-coding genes, 2 rRNA genes, and 22 tRNA genes. The overall G + C proportion is 31.48%. These data expand the termite mitogenome databases, provide data support for the next phylogenetic and evolutionary studies.

West Indian drywood termite, *Cryptotermes brevis* (Walker) (Isoptera: Kalotermitidae), is an important urban pest and invasive species (Constantino 2002). *Cryptotermes brevis* originated in the Pacific coastal desert of South America and spread widely in the tropical and subtropical areas (Nunes et al. 2010; Ferreira and Teresa 2011). It causes damage to sheltered wood products, such as furniture, door frames, floor, even wooden carvings, photo frames, musical instruments (Gay and Watson 1982; Constantino 2002; Scheffrahn et al. 2009; Ferreira and Teresa 2011). Here, we report the complete mitochondrial genome sequences of *C. brevis*, which will provide data support for the next phylogenetic and evolutionary studies.

Specimens were collected from Qingdao of Shandong Province, China, and kept in the Laboratory of Invasion Biology, College of Agriculture, Jiangxi Agricultural University, Jiangxi, China. The circular mitogenomes of *C. brevis* is 15,674 bp in size (Genbank: MK618724). The mitochondrial genome contain 13 protein-coding genes (PCGs), 22 transfer RNA (tRNA) genes, and 2 ribosomal RNA (rRNA) genes. The complete mitochondrial genome sequences of *C. brevis* consisted of 42.66% A, 19.89% C, 11.59% G, and 25.86% T. The overall A + T content of *C. brevis* is 68.52%, showing obvious A + T biase. The protein-coding genes (PCGs) have a total length of 10,985 bp. The size of the two rRNAs are 1374 bp and 808 bp, respectively.

The majority strand (J-strand) codes 23 genes including 9 PCGs (nad2, cox1-3, atp6, atp8, nad 6, nad 3, and cob) and 14 tRNA genes (trnI, trnM, trnW, trnL2, trnK, trnD, trnG, trnA, trnR, trnN, trnS1, trnE, trnT and trnS2). The other 14 genes were coded by the minority strand (N-strand). All of the 22 tRNAs, ranging from 61 to 72 bp, have a classical clover leaf structures, except for trnH and trnY lacking the TψC loop and trnS1 lacking the DHU arm.

Based on the reported data of 11 species of termites, we combined 13 PCGs and 24RNAs (22tRNAs, 2RNAs) to construct MrBayes phylogenetic trees which is shown in Figure 1 (Ronquist et al. 2012; Katoh and Standley 2013; Lanfear et al. 2016; Zhang et al. 2018). This result was similar to the previous molecular studies of Kalotermitidae (Liao et al. 2018).

**Figure 1.**
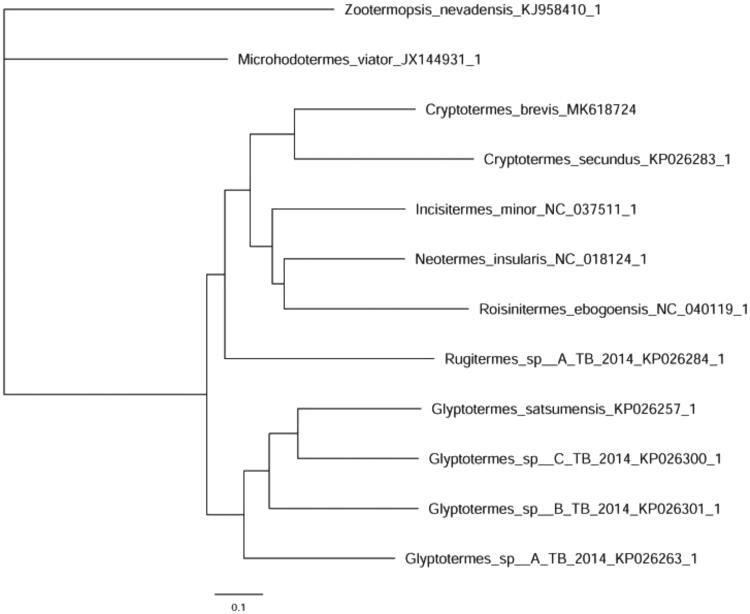
MrBayes phylogenetic tree of selected termite mitogenomes including Kalotermitidae. The phylogenetic tree was built using all protein-coding genes (PCGs) and RNAs. *Microhodotermes viator* and *Zootermopsis nevadensis* were used as outgroups. GenBank accession numbers are given after the species name.
